# Tuning the H‐Atom Transfer Reactivity of Iron(IV)‐Oxo Complexes as Probed by Infrared Photodissociation Spectroscopy

**DOI:** 10.1002/anie.202016695

**Published:** 2021-02-17

**Authors:** Guilherme L. Tripodi, Magda M. J. Dekker, Jana Roithová, Lawrence Que

**Affiliations:** ^1^ Department of spectroscopy and Catalysis Institute for Molecules and Materials Radboud University Nijmegen Heyendaalseweg 135 6525 AJ Nijmegen The Netherlands; ^2^ Department of Chemistry University of Minnesota Minneapolis Twin Cities 207 Pleasant Street SE 55455 MN USA

**Keywords:** C−H activation, ion spectroscopy, iron-oxo, mass spectrometry, reactivity screening

## Abstract

Reactivities of non‐heme iron(IV)‐oxo complexes are mostly controlled by the ligands. Complexes with tetradentate ligands such as [(TPA)FeO]^2+^ (TPA=tris(2‐pyridylmethyl)amine) belong to the most reactive ones. Here, we show a fine‐tuning of the reactivity of [(TPA)FeO]^2+^ by an additional ligand X (X=CH_3_CN, CF_3_SO_3_^−^, ArI, and ArIO; ArI=2‐(^*t*^BuSO_2_)C_6_H_4_I) attached in solution and reveal a thus far unknown role of the ArIO oxidant. The HAT reactivity of [(TPA)FeO(X)]^+/2+^ decreases in the order of X: ArIO > MeCN > ArI ≈ TfO^−^. Hence, ArIO is not just a mere oxidant of the iron(II) complex, but it can also increase the reactivity of the iron(IV)‐oxo complex as a labile ligand. The detected HAT reactivities of the [(TPA)FeO(X)]^+/2+^ complexes correlate with the Fe=O and FeO−H stretching vibrations of the reactants and the respective products as determined by infrared photodissociation spectroscopy. Hence, the most reactive [(TPA)FeO(ArIO)]^2+^ adduct in the series has the weakest Fe=O bond and forms the strongest FeO−H bond in the HAT reaction.

## Graphical abstract

The reaction kinetics of H‐atom transfer mediated by iron(IV)oxo complexes with different cis‐ligands can be studied in a modular flow reactor coupled to MS detection. The HAT reactivity correlates with the intrinsic properties of the isolated complexes in the gas phase. A ligand with a larger binding energy forms a more reactive complex for the HAT reactions, has a weaker Fe^I*V*^=O bond, and forms a stronger Fe^III^O−H bond after the HAT reaction.

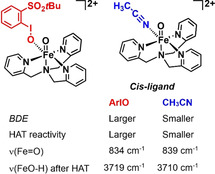

## Introduction

The development of non‐heme iron(IV)‐oxo complexes has been inspired by enzymatic oxidations.^[1]^ Enzymes working with non‐heme iron(IV)‐oxo reactive forms can oxidize inert C−H bonds in a selective fashion.^[2]^ The efforts on synthetic non‐heme iron complexes have focused on understanding the ligand effect on the properties and reactivity of the generated reactive iron(IV)‐oxo species.^[3]^

**Scheme 1:**
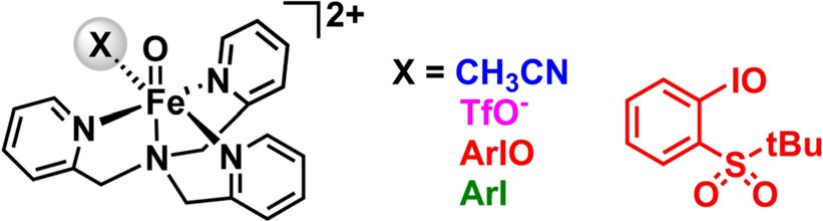
Iron(IV)‐oxo complexes formed by oxidation of [(TPA)Fe^II^(TfO)_2_] with ArIO (ArI=2‐(^t^BuSO_2_)C_6_H_4_I).

A prototypical complex in this field is one supported by the tetradentate tripodal ligand TPA (tris(2‐pyridylmethyl)‐amine).^[4]^ It has been shown that [(TPA)Fe^II^(TfO)_2_] (TfO^−^=trifluoromethylsulfonate or triflate) can be oxidized by peracids or iodosobenzene (or its derivatives) in acetonitrile to form a fairly reactive [(TPA)Fe^IV^O(MeCN)]^2+^ complex (Scheme 1).^[5]^ Subsequent modifications of the TPA ligand have led to even more reactive complexes.^[6]^

The common feature of the [(L)Fe^IV^(O)]^2+^ complexes (L=tetradentate tripodal ligand) is that a solvent molecule or another 2 e^−^ donor ligand is often attached as the sixth ligand (unless the supporting ligand is sterically hindered).^[7]^ This labile ligand offers another opportunity to tune the reactivity of this type of iron(IV)‐oxo complexes, although this aspect has thus far not been investigated in that much detail.^[7]^

In solution, we expect an equilibrium among complexes with different labile ligands. The observed bulk reactivity then reflects the average of the reactivities of these different complexes weighted by their abundance. Here, we show an approach that allows different forms of reactive complexes to be detected and their reactivity to be compared. For this study, we focus on a family of iron(IV)‐oxo complexes [(TPA)Fe^IV^O(X)]^2+^ (X=labile ligand, Scheme 1). In addition, this family of analogous complexes offer an ideal platform to evaluate how the properties of the Fe=O bond and the newly formed FeO−H bond affect the reactivities of these complexes in HAT reactions.

## Results and Discussion

**Figure 1:**
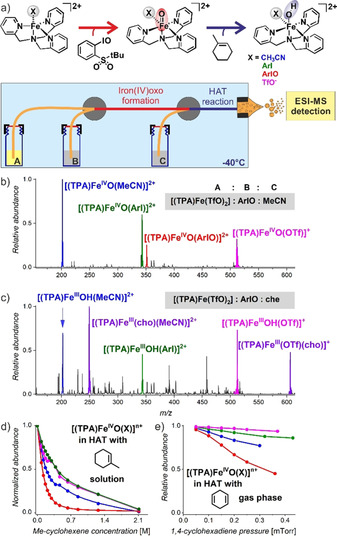
a) Generation and reaction of iron(IV)‐oxo complexes in a flow reactor with ESI‐MS detection. The flow is secured by nitrogen overpressure in the vials (not shown, see Figure S3) b) Detection of iron(IV)‐oxo complexes (vial C contained only acetonitrile). c) Detection of products of the HAT reaction with 1‐methylcyclohexene (che) (cho=deprotonated 3‐methylcyclohex‐2‐en‐1‐ol). The concentrations of the solutions were: [(TPA)Fe(TfO)_2_] (1 mM), ArIO (1.4 mM), and 1‐methylcyclohexene (1 M). d) The relative abundance of the iron(IV)‐oxo complexes in reaction with 1‐methylcyclohexene as a function of increasing concentration of 1‐methylcyclohexene. e) Pressure dependence of the reactivity between [(TPA)Fe^IV^O(X)]^+/2+^ complexes and 1,4‐cyclohexadiene, see Figures S5–7 for further details.

Mass spectrometry has been an established method in the field for detection of reactive iron(IV)‐oxo complexes.^[8]^ Most often, researchers use the cryospray method at −40 °C to transfer the reactive complexes to the gas phase.^[9]^ In this study, we prepare the reactive complexes in a flow reactor from a silica capillary coupled to the electrospray ionization chamber, thereby shortening the time for the oxidation reaction (Figure 1 a). The whole setup is cooled to −40 °C and the reaction time (*t*_r_) in the flow is typically about 10 seconds.

### Oxidation of Iron(II) Complexes

Charging the vial A with an acetonitrile solution of [(TPA)Fe^II^(TfO)_2_] (vials B and C contain only acetonitrile) leads to the detection of the expected iron(II) complexes [(TPA)Fe^II^(MeCN)]^2+^ and [(TPA)Fe^II^(TfO)]^+^ (Figure S4). Addition of the oxidant 2‐(^t^BuSO_2_)C_6_H_4_IO (ArIO) to vial B results in oxidation of the iron(II) complexes and detection of [(TPA)Fe^IV^O]^2+^, [(TPA)Fe^IV^O(MeCN)]^2+^, and [(TPA)Fe^IV^O(TfO)]^+^ ions, in analogy to previously reported results (Figure 1 b).^[5]^ In addition, the low temperature and short reaction time allow the detection of [(TPA)Fe^IV^O(ArIO)]^2+^ and [(TPA)Fe^IV^O(ArI)]^2+^ ions, corresponding to signals of iron(IV)‐oxo complexes, respectively bound to the oxidant itself as well as the oxidation byproduct ArI.

### Hydrogen Atom Transfer (HAT)

The reaction of the iron(IV)‐oxo complexes that is of most interest is hydrogen atom transfer with hydrocarbons, which we have studied with 1‐methylcyclohexene (che, Figure 1 c). After adding the hydrocarbon to the vial C, we can monitor the HAT reaction proceeding in solution before ESI‐MS detection. The reaction conversion can be tuned by changes in the concentration of the hydrocarbon (vial C). The products are iron(III)‐hydroxo species (Figure 1 c) and iron(III) alkoxide complexes bearing the deprotonated hydroxylated product 3‐methylcyclohex‐2‐en‐1‐ol (cho, see Figure S8 for fragmentation patterns). Note that the HAT reaction proceeds in solution in the flow reactor as evidenced by the conversion changes depending on the reaction time (i.e., the capillary length, see Figure S5).

In order to compare the kinetics of the reactions of the [(TPA)Fe^IV^O(X)]^+/2+^ complexes, we have systematically varied the concentration of 1‐methylcyclohexene in vial C (Figure 1 d). With the increasing concentration of the alkene, we have observed a larger conversion of iron(IV)‐oxo complexes to the iron(III) products. From the relative abundances of the [(TPA)Fe^IV^O(X)]^+/2+^ complexes as detected by ESI‐MS, we conclude that [(TPA)Fe^IV^O(ArIO)]^2+^ reacts the fastest, followed by [(TPA)Fe^IV^O(MeCN)]^2+^ and finally by [(TPA)Fe^IV^O(ArI)]^2+^ and [(TPA)Fe^IV^O(TfO)]^+^ with almost identical rates (Figure 1 d). A more quantitative assessment of the data is hampered by the fact that the curves do not only reflect the HAT reactivity, but also the equilibrium among the [(TPA)Fe^IV^O(X)]^+/2+^ complexes.

The relative HAT reactivities of the isolated [(TPA)Fe^IV^O(X)]^+/2+^ complexes can be compared exactly in gas phase experiments. In the gas phase, we had to use the more reactive 1,4‐cyclohexadiene as the substrate (Figure 1 e). Indeed, [(TPA)Fe^IV^O(ArIO)]^2+^ exhibits the fastest rate, followed by [(TPA)Fe^IV^O(MeCN)]^2+^, with [(TPA)Fe^IV^O(ArI)]^2+^ and [(TPA)Fe^IV^O(TfO)]^+^ having the slowest rates. The gas phase experiments provide quantitatively correct rate constants and the relative HAT rates of [(TPA)Fe^IV^O(X)]^+/2+^ ions decrease in the order of X as ArIO/MeCN/ArI/TfO^−^=1:0.45:0.08:0.07.

Our results show that the thus far never considered complex [(TPA)Fe^IV^O(ArIO)]^2+^ is more reactive than the usually considered [(TPA)Fe^IV^O(MeCN)]^2+^. However, the importance of these complexes for the observed oxidation reaction depends on their relative abundance in solution. The equilibrium between the individual [(TPA)Fe^IV^O(X)]^2+^ complexes depends on the concentration of X and on the relative free energies of the [(TPA)Fe^IV^O(X)]^+/2+^ complexes in solution.

First, we have assessed the relative binding energies of X in [(TPA)Fe^IV^O(X)]^2+^ obtained from theoretical calculations. Theory predicts that the binding energies of X increase in the order: CH_3_CN (*BDE*=8.9 kcal mol^−1^) < ArI (Δ*BDE*=10.4 kcal mol^−1^) ≈ TfO^−^ (Δ*BDE*=12.6 kcal mol^−1^) ≪ ArIO (Δ*BDE*=25.0 kcal mol^−1^). For comparison, we have studied the trend of the binding energies experimentally in the gas phase, which is consistent with these theoretical values (Figure S9). The entropic effects in solution disfavor the stability of [(TPA)Fe^IV^O(ArI)]^2+^ leading to the predicted trend in the equilibrium reactions shown in Table 1. Combination of these predicted free energies with the concentrations of the ligands in the solution suggests that [(TPA)Fe^IV^O(ArIO)]^2+^ should be formed by far preferentially. Note, that we did the experiments with 1.4 equiv of ArIO. Hence only ≈0.4 equiv ArIO is available for the formation of [(TPA)Fe^IV^O(ArIO)]^2+^. The only other iron(IV)oxo complex will be present as [(TPA)Fe^IV^O(MeCN)]^2+^ (Table 1 and Table S3). The [(TPA)Fe^IV^O(X)]^2+^ complexes with X=TfO^−^ or ArI and their role for the reactivity in solution can be neglected.

**Table 1:** Relative free energies^[a]^ for the equilibrium reactions in acetonitrile and the respective relative rate constants for the formation of the iron(IV)oxo complexes.

Complexes in equilibrium	ΔΔ*G* ^298K^ [kcal mol^−1^]	*k*′_rel_^[b]^
[(TPA)Fe^IV^O(MeCN)]^2+^ + ArI + TfO^−^ + ArIO	0	1
[(TPA)Fe^IV^O(TfO^−^)]^+^ + ArI + MeCN + ArIO	−2.0	8.10^−3^
[(TPA)Fe^IV^O(ArI)]^2+^ + MeCN + TfO^−^ + ArIO	2.3	4.10^−7^
[(TPA)Fe^IV^O(ArIO)]^2+^ + ArI + TfO^−^ + MeCN	−12.7	2.10^7^

[a] The calculations were performed at the B3LYP^[11]^‐D3^[12]^/def2tzvp^[13]^ level using SMD^[14]^ to account for the solvation by acetonitrile. [b] The relative rate constants are multiplied by concentrations of the free ligands in the solution assuming the complete conversion in oxidation of 1 mM [(TPA)Fe(TfO)_2_] by 1.4 equiv of ArIO in acetonitrile ([TfO^−^]=2 mM, [ArI]=1 mM, [ArIO]=0.4 mM, [MeCN]=19 M).

### UV/Vis Spectroscopy

**Figure 2:**
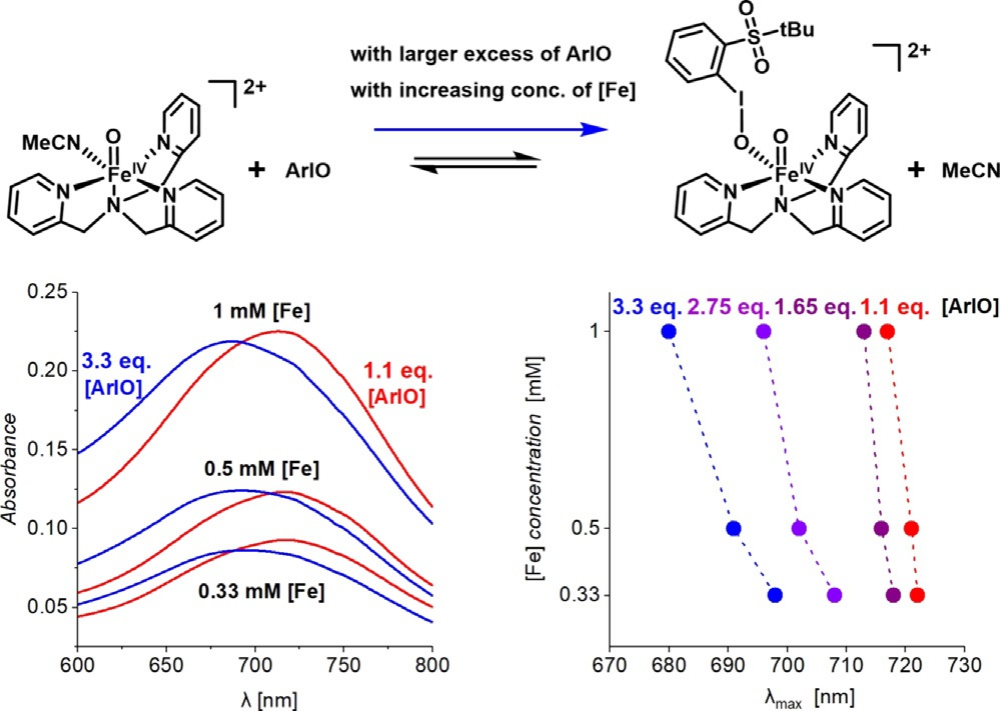
Left panel: electronic absorption spectra for the reaction between [(TPA)Fe(TfO)_2_] and ArIO at different concentrations of [(TPA)Fe(TfO)_2_] (denoted as [Fe]) and in the presence of 1.1 equiv (red) or 3.3 equiv (blue) of ArIO with respect to the concentration of the iron complex. Right panel: *λ*_max_ of the iron(IV)‐oxo complex as a function of the concentration of [(TPA)Fe(TfO)_2_] and the amount of ArIO added for the oxidation of [(TPA)Fe(TfO)_2_]. Red is for 1.1 equiv. of ArIO, purple for 1.65 equiv., violet for 2.75 equiv., and blue for 3.3 equiv of ArIO.

We have tested the hypothesis that [(TPA)Fe^IV^O(ArIO)]^2+^ is preferentially formed in solution by UV/Vis spectroscopy. The parent *S*=1 complex [(TPA)Fe^IV^O(MeCN)]^2+^ exhibits a maximum absorbance (*λ*_max_) at 722 nm when generated by oxidation with peracetic acid.^[4]^ The maximum slightly blue‐shifts when the iron‐oxo species is generated by oxidation with an excess of ArIO (Figure 2).^[5]^ Previous studies showed that *λ*_max_ is sensitive to the nature of the *cis*‐ligand.^[15]^ Hence, the blue shift observed with ArIO oxidation can be a result of ArIO coordination to a position *cis* to the Fe=O unit to generate [(TPA)Fe^IV^O(ArIO)]^2+^.

As explained above, we consider only the equilibrium between [(TPA)Fe^IV^O(CH_3_CN)]^2+^ and [(TPA)Fe^IV^O(ArIO)]^2+^ in solution. Hence, with increasing concentration of ArIO, a larger fraction of the iron(IV)‐oxo complexes should be converted to [(TPA)Fe^IV^O(ArIO)]^2+^, accounting for the observed increase in the blue shift. On the other hand, diluting the solution should contribute to the solvolysis of the complex (Figure 2, note that in this experiment we work under equilibrium conditions unlike in the ESI‐MS experiments). Consistent with our postulate, we observe a red shift of *λ*_max_, when we dilute the reaction mixture, and a significant blue shift, when we increase the relative concentration of ArIO. We observe the previously reported *λ*_max_ at 722 nm, only if 1.1 equiv. of ArIO is added to the most dilute solution studied (0.33 mM). Hence, we can conclude that [(TPA)Fe^IV^O(CH_3_CN)]^2+^ is the prevailing product under these conditions. However, adding more equivalents of ArIO and working at larger concentrations lead to generation of the more reactive [(TPA)Fe^IV^O(ArIO)]^2+^ complex.

### Correlation of Reactivity and Stretching Frequencies of the Fe^IV^=O and Fe^III^O−H Bonds

The family of [(TPA)Fe^IV^O(X)]^+/2+^ complexes constitutes an ideal system to test to what extent the relative reactivities correlate with properties of the reactive Fe=O bonds and with the newly formed FeO−H bonds. According to the Bell–Evans–Polanyi principle,^[16]^ we can roughly predict relative rates of HAT reactions based on the reaction enthalpies [Eq. 1].

(1) $$\mathrm{Fe}{}^{\mathrm{IV}}=O\;+\;R-H\;\to \;\mathrm{Fe}{}^{\mathrm{III}}-\mathrm{OH}\;+\;R{}^{•}\Delta \Delta H{}^{\mathrm{HAT}}\approx \Delta H{}_{\mathrm{FeO}-H}-\Delta H{}_{\mathrm{Fe}=O}$$

Mayer^[17]^ and Shaik^[18]^ have shown that the barrier for HAT reactions can be estimated based on the bond dissociation energy of the newly formed O−H bond (*BDE*_OH_).^[17e]^ However, it is difficult to assess the *BDE*s of FeO−H bonds experimentally, therefore the studies rely on theoretical data. To date, the experimental *BDE*_OH_ values of only two non‐heme Fe^III^OH complexes have been obtained, but these values have been determined only by indirect means.^[19]^ In principle, the Fe=O and FeO−H bonds can be studied directly by vibrational spectroscopy. Stretching frequencies of the bonds correlate with their strength but, so far, it was impossible to obtain vibrational information on the FeO−H bonds of the product complexes. In the following, we demonstrate that infrared photodissociation spectroscopy can easily carry out direct experimental measurements of the bonds of interest and shed light on a critical question in this field.^[20]^

### Infrared Characteristics of [(TPA)Fe^IV^O(X)]^+/2+^ and [(TPA)Fe^III^(OH)(X)]^+/2+^

**Figure 3:**
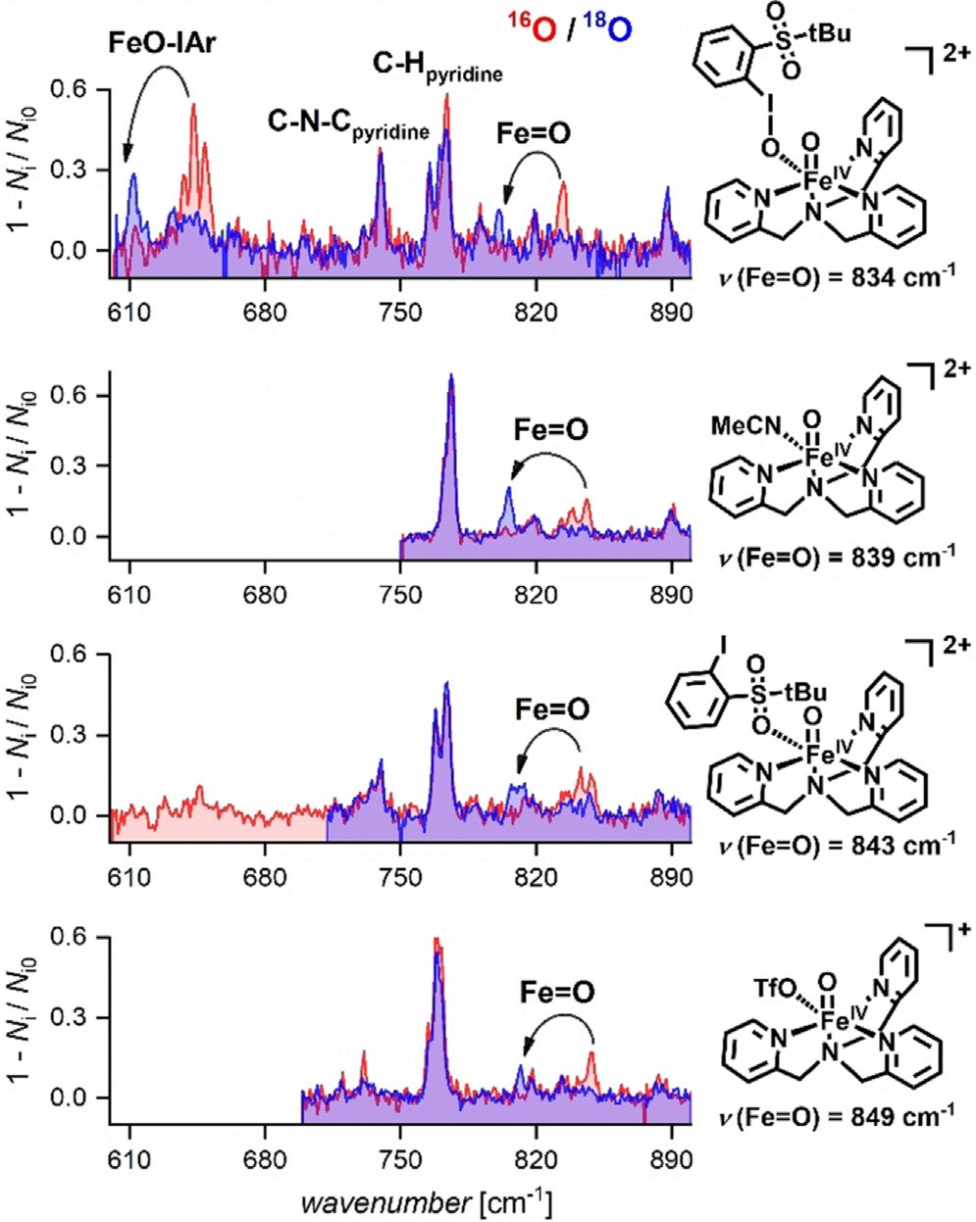
IRPD spectra for [(TPA)Fe^IV^O(X)]^+2+^ (L=ArIO, ArI, MeCN and TfO^−^) (in red) and for analogous complexes generated with ArI^18^O (in blue).

Helium‐tagging infrared photodissociation (IRPD) spectroscopy allows the measurement of IR spectra of mass‐selected ions.^[21]^ The Fe=O stretching frequencies of all four detected [(TPA)Fe^IV^O(X)]^+/2+^ complexes can readily be assigned based on their ^16^O/^18^O shifts. We observe that the Fe=O stretching frequencies of these four [(TPA)Fe^IV^O(X)]^+/2+^ complexes decrease in the following order: 849 cm^−1^ for X=TfO^−^, 843 cm^−1^ for X=ArI, 839 cm^−1^ for X=ACN, and 834 cm^−1^ for X=ArIO, (Figure 3, Table 2). For [(TPA)Fe^IV^O(ArIO)]^2+^, there is also a feature at about 640 cm^−1^ that is sensitive to ^18^O labelling and most likely arises from the I=O stretching vibration of the ArIO ligand.

**Table 2:** Fe=O stretching frequencies in [(TPA)Fe^IV^O(X)]^+/2+^ and O−H stretching frequencies in [(TPA)Fe^III^(OH)(X)]^+/2+^.

X	*v*(Fe=O) [cm^−1^]		*v*(O−H) [cm^−1^]	
	*IRPD* ^[a]^	*DFT* ^[b]^	*IRPD* ^[a]^	*DFT* ^[c]^
ArIO	834±2	841 (934)	3719±2	3697 (3851)
MeCN	839±2	823 (914)	3710±2	3704 (3858)
ArI	843±2	839 (932)	3645±2	3704 (3858)
TfO^‐^	849±2	840 (933)	3643±2	3613 (3764)

[a] The error refers to the accuracy of the measurement and evaluation of the data. The relative error is much smaller. [b] The B3LYP‐D3/def2tzvp frequencies scaled by 0.9. The unscaled frequencies are in the brackets. [c] The B3LYP‐D3/def2tzvp frequencies scaled by 0.96. The unscaled frequencies are in the brackets.

**Figure 4:**
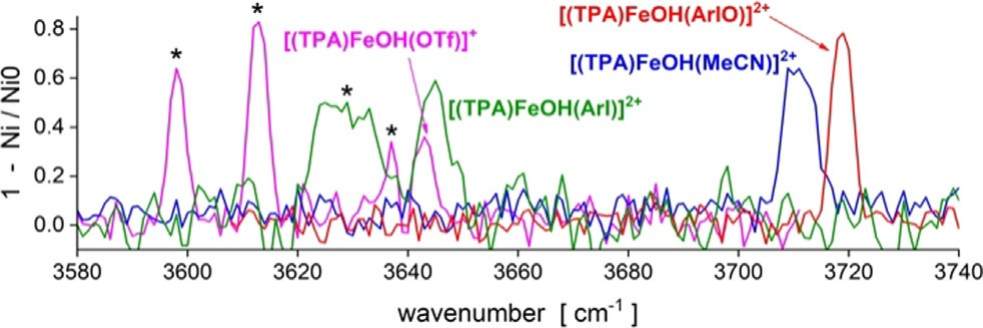
IPRD spectra for [(TPA)Fe^III^OH(X)]^+/2+^ (X=ArIO in red, ArI in green, MeCN in blue and TfO^−^ in pink). The bands with asterisks * represent O−H bands hydrogen bonded to the respective ligands X (see Figure S11).

Similarly, we have determined the stretching frequencies of the O−H bond in the [(TPA)Fe^III^(OH)(X)]^+/2+^ complexes (Figure 4). The [(TPA)Fe^III^OH(ArI)]^2+^ and [(TPA)Fe^III^OH(TfO)]^+^ complexes show several O‐H bands, which is due to the possibility of internal hydrogen bonding between the O‐H group and the oxygen atoms of the SO_*x*_ moiety at ArI or triflate. This interaction leads to a red shift of the respective O‐H vibrations. We have optimized several conformers that rationalize the presence of several red‐shifted bands (Figure S11 in the SI). For further discussion, we have focused on the highest lying O‐H band for each complex, because this vibration should correspond to the free O‐H vibration.

Inspection of the O‐H vibrations reveals that the iron(IV)‐oxo complexes with lower Fe^IV^=O frequencies generate Fe^III^‐hydroxo complexes with higher Fe^III^O‐H vibrational frequencies. Notably, there is a much larger spread in the stretching energies for the Fe^III^O−H bonds than for the Fe=O bonds, emphasizing the higher sensitivity of these bonds to changes in their ligand environments.

The measured stretching frequencies of the reacting and the newly formed bonds predict that the HAT reactivities of [(TPA)Fe^IV^O(X)]^+/2+^ should follow the order of X: ArIO > MeCN > ArI ≈ TfO^−^. This trend is the same as the trend observed experimentally. It shows that it is valid to approximately evaluate the reactivities of iron(IV) oxo complexes based on the properties of the Fe^IV^=O and the Fe^III^O−H bonds.

However, the trends of the Fe=O and FeO‐H stretching frequencies are not reproduced by the DFT calculations (Tables 2, S1 and S2, Figure S12). According to these calculations, [(TPA)Fe^IV^O(MeCN)]^2+^ has the weakest Fe=O bond and [(TPA)Fe^IV^O(TfO)]^+^ forms the strongest O−H bond. Strikingly, the most reactive [(TPA)Fe^IV^O(ArIO)]^2+^ complex would be predicted as the least reactive based on the theoretical Fe=O and FeO‐H stretching frequencies. These contradicting trends point to the limits of DFT in describing this type of complexes. There is an ample of examples that DFT describes correctly reactivity and properties of various hypervalent metal complexes. However, there might be a limitation when going towards description of subtle differences such as the effect of labile cis‐ligands as described here. Hence, we believe that our results show the need for experimental data that describe properties and reactivity trends of these complexes to benchmark the theoretical methods.

## Conclusion

We have investigated the formation and hydrogen‐atom transfer (HAT) reactivity of [(TPA)Fe^IV^(O)(X)]^+/2+^ (X=MeCN, TfO^−^, ArI, ArIO) complexes in a flow setup with electrospray ionization mass spectrometry (ESI‐MS) detection. We show that we can investigate solution reaction kinetics and employ sensitive ESI‐MS detection at the same time. This approach has allowed us to show that the HAT reactivity of [(TPA)Fe^IV^(O)(X)]^+/2+^ with 1‐methylcyclohexene decreases in the order of X: ArIO > MeCN > ArI ≈ TfO^−^. The theory predicts that the binding energy of ArIO to the iron core is about 16 kcal mol^−1^ larger than that of MeCN and that the formation of [(TPA)Fe^IV^(O)(ArIO)]^2+^ in solution should be largely preferred if an excess of ArIO is used in the experiment. The importance of [(TPA)Fe^IV^(O)(ArIO)]^2+^, a species which to date has not been considered to be a participant in this chemistry, is further supported by UV/Vis spectroscopy.

Furthermore, we have evaluated the effect of the ligand X on the Fe^IV^=O and the Fe^III^O−H bonds in the [(TPA)Fe^IV^(O)(X)]^+/2+^ and [(TPA)Fe^III^(OH)(X)]^+/2+^ complexes, respectively. The Fe=O stretching frequency increases in the order of X: ArIO < MeCN < ArI < TfO^−^, while that of the FeO‐H unit decreases in the same order. Hence a weaker Fe=O bond gives rise to a stronger FeO−H bond, which in turn facilitates the HAT reaction. This trend directly correlates with the measured HAT activities of these complexes and demonstrates in particular the role of the O‐H formation in serving as a driving force for HAT reactions, as postulated by Shaik^[18]^ and Mayer.^[17]^

## Conflict of interest

The authors declare no conflict of interest.

## Supporting information

As a service to our authors and readers, this journal provides supporting information supplied by the authors. Such materials are peer reviewed and may be re‐organized for online delivery, but are not copy‐edited or typeset. Technical support issues arising from supporting information (other than missing files) should be addressed to the authors.

Supplementary
