# Supplementary material for: Tuning the H‐Atom Transfer Reactivity of Iron(IV)‐Oxo Complexes as Probed by Infrared Photodissociation Spectroscopy
Source: Angew Chem Int Ed Engl. 2021 Feb 17;60(13):7126–31. doi: 10.1002/anie.202016695 (PMC8048595; doi:10.1002/anie.202016695)
Supplement: Supplementary file 1 — Supplementary [file ANIE-60-7126-s001.pdf]

Angewandte Chemie
Eine Zeitschrift der Gesellschaft Deutscher Chemiker

Supporting Information

Tuning the H-Atom Transfer Reactivity of Iron(IV)-Oxo Complexes as Probed by Infrared Photodissociation Spectroscopy

Guilherme L. Tripodi, Magda M. J. Dekker, Jana Roithová, and Lawrence Que, Jr.**

anie_202016695_sm_miscellaneous_information.pdf

Table of Contents

Experimental Procedures S2
Chemical synthesis S2
UV-Vis experiments S2
Electrospray mass spectrometry experiments..... S2
Experiments in flow-setup S3
Kinetics in solution S3
Helium-tagging photo dissociation spectroscopy S3
Computational details S4
Results S4
Reactivity in solution S4
Gas phase reactivity S5
Collision Induced dissociation experiments with mass selected ions..... S6
Infrared photodissociation spectra S7
DFT calculations S8
XYZ coordinates from DFT calculations..... S9
Supplementary References S19

Experimental Procedures

Chemical synthesis. The chemicals 2-(tert- butylsulfonyl)iodosylbenzene[S1] (2-(tBuSO2)$C_6H_4IO)_2$ and [(TPA)Fe(OTf)$_2$][S2] were prepared according to the published procedures.

UV-Vis experiments. UV-vis spectra were recorded on JASCO V-630 UV-Vis spectrometer equipped with a thermostatted cell holder. Experiments were performed at 0^0C.

Electrospray mass spectrometry experiments. The experiments were performed with triple quadrupole mass spectrometer TSQ Classic equipped with an electrospray ionization source.[S3] The complexes were transmitted from solution to the gas phase at soft ionization conditions and mass-analyzed by the first quadrupole (the octopole and the second quadrupole were in total transmission mode). The ions are detected by a conversion dynode and electron multiplier. Electrospray conditions typically were: 4 kV spray voltage, 50 ^{0}C capillary temperature, 0 V capillary voltage, 40 V tube lens voltage, 40 psi of N_2 sheath gas. The use capillary temperature above 50 ^{0}C leads to decomposition of the iron-oxo complexes via ligand oxidation. This is observed by the lower gas phase reactivity and distinct IR signatures when higher temperatures were used (Figure S1).

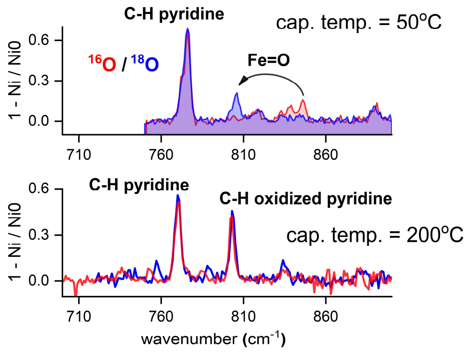

Figure S1. IRPD spectra of mass selected ions of *m/z* 201.5 (red) and 202.5 (blue) generated via mixing of [(TPA)Fe(OTf)$_2$] with ArI^{16}O and ArI^{18}O, respectively. The top spectrum was recorded with capillary temperature at 50 ^{0}C and the bottom spectrum at 200 ^{0}C.

The MS/MS measurements were performed for ions mass-selected by the first quadrupole. These ions were collided at a specified energy with either xenon (collision induced dissociation, CID) or with an organic reactant (gas-phase reactivity studies). The products of the collisions were mass-analyzed by the second quadrupole and detected by a dynode/multiplier system. The pressure in the collision cell was measured with 120 AA Baratron (MKS intruments). All CID experiments were performed with 0.15 mTorr pressure of Xe in the collision cell. The pressure of organic reactants were varied and are specified for each experiment. The zero collision energy was determined by retarding potential analysis (Figure S2). Energies were converted from E_{lab} (laboratory energy) to E_{CM} (center-of-mass energy) by the formula $E_{CM} = E_{lab}\, m(m + M)$, where m and M are the masses of the reactant neutral (xenon) and the ion, respectively. The reactivity with organic reactants was always studied at zero collision energy.

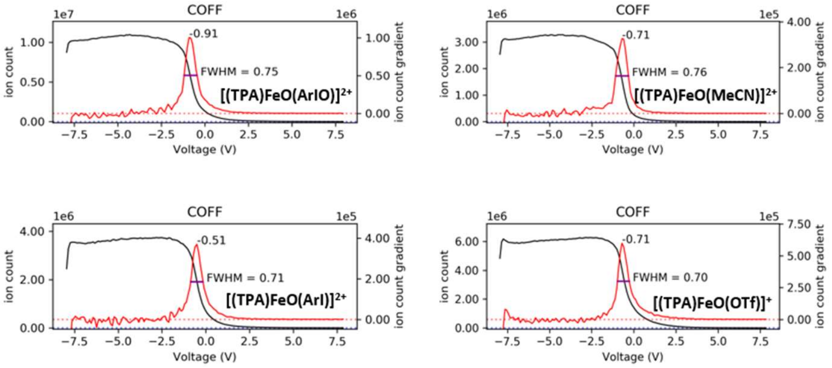

Figure S2. Kinetic energy distribution of $[(TPA)FeO(X)]^{n+}$ measured by the retarding potential analysis. Black trace is the ion current, whereas the red trace corresponds to the energy distribution (first derivative of the ion current in respect to the collision offset).

Experiments in a flow setup. Ions were generated in a home-made flow reactor made of silica capillaries and polypropylene mixing-Ts as depicted in Figure S3 and transferred to the gas phase via electrospray. Sequential mixing of solutions was performed by in series connection of mixing Ts (Figure S3). The flow-rate is controlled by applying an overpressure of N2 of approximately 2 psi into each vial. In order to estimate the residence time, the silica capillary that is inserted into the solution is made much shorter than the capillary in which the reaction takes place. As soon as the vial with the sample solution is inserted into the flow reactor, we start to record the mass spectrum. The residence time is estimated to be the time needed for the signals of the new sample to appear on the mass spectrum.

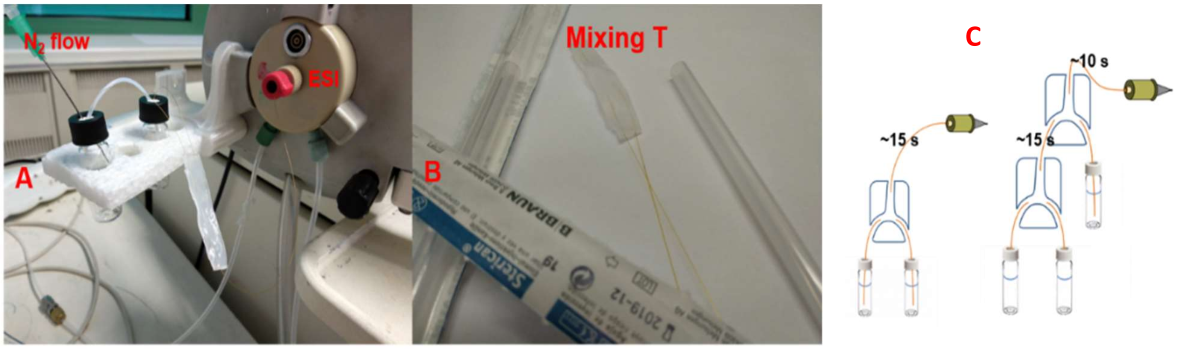

Figure S3 A) Real picture of home-made flow reactor with one mixing T directly connect to the electrospray (ESI) source of the mass spectrometer. A flow of N_2 is applied to one of the vials and a connector between the vials equilibrates their pressure. B) Real picture of mixing T made from the polypropylene tube of 8 cm needle from Sterican Safety Needles – B/BRAUN. C) Schematic representation of flow-setups.

Kinetics in solution. Kinetics for the reaction between $[(TPA)FeO(X)]^{n+}$ and Me-cyclohexene were measured in a flow reactor with 2 mixing Ts directly connected to the electrospray source of the TSQ 7000 mass spectrometer. A constant flow of N_2 was applied into the first two vials containing $[(TPA)Fe(OTf)_2]$ and ArIO. A connector between these first two vials is placed in order to maintain the same pressure on both of them (Figure S3-A). A second individual flow of N_2 is placed into the third vial. The content of the third vial was changed to the desired concentrations of Me-cyclohexene and mass spectra was recorded for at least 4 minutes at each concentration. The intensities of the ions $[(TPA)FeO(X)]^{n+}$ was normalized by the total ion chromatogram (TIC) and plotted as a function of the concentration of Me-cyclohexene on the third vial.

Helium-tagging photodissociation spectroscopy. The mass-selected $[(TPA)FeO(X)]^{n+}$ ions were analyzed by infrared photodissociation spectroscopy using the ISORI instrument and the helium-tagging method at 3 Kelvin as described earlier.[S4] We monitored the light-induced dissociation of weakly-bound complexes of studied ions with helium. This was achieved by trapping the mass-selected ions in the cryogenic ion trap with a helium pulse, which resulted in formation of helium complexes. These complexes were extracted from the ion trap after being stored for a specified time (1–10 seconds) and counted to obtain their reference count N_{i0}. In the alternative trapping cycle, the ion cloud was irradiated with a laser light during the storage time. This destroyed some of the labile helium complexes and a lower helium complexes count Ni was obtained after the extraction from the trap. The infrared photodissociation (IRPD) spectra were acquired by recording the dissociation yield of the helium complexes (attenuation), defined as 1 - N_i / N_{i0}, while scanning the laser frequency.

Computational details. DFT calculations were performed at the B3LYP-D3/Def2TZVP level of theory using Gaussian 16 package. SDM solvation model was applied in order to account for acetonitrile solvation. All reported structures correspond to minima on the potential energy surface as confirmed by analyses of the corresponding Hessian matrixes. Reported energies include zero-point vibrational energy correction and thermal corrections calculated at the same level. The molecular coordinates are provided in the end of this Supporting Information.

Results

Reactivity in solution

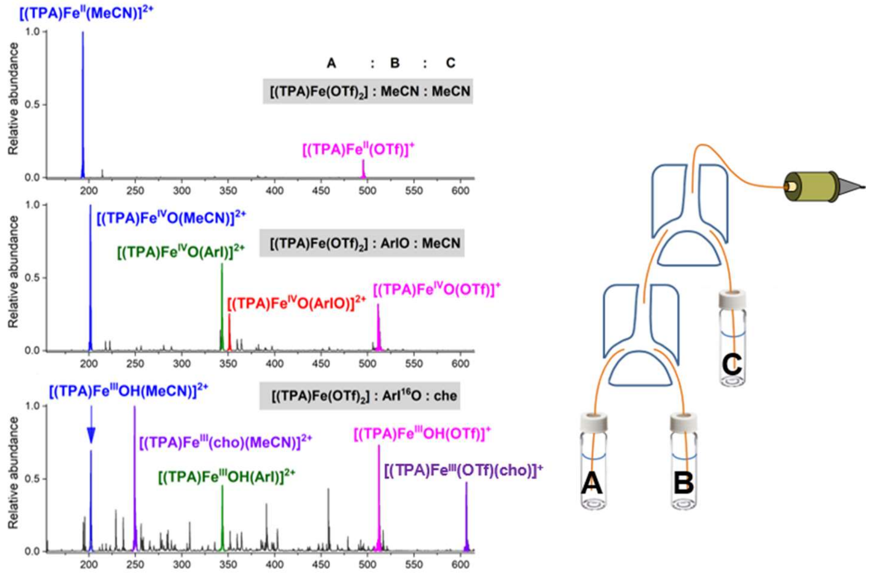

Figure S4. A sketch of a silica-capillary flow reactor connected to an electrospray ionization head of a mass spectrometer and ESI-MS spectra of reaction mixtures resulting from sequential mixing of solutions in vials A – C; the flow was constant. The content of the vials is specified at each spectrum. The abbreviations che, cho stand for 1-methylcyclohexene and deprotonated 3-methylcyclohex-2-en-1-ol, respectively. The relative concentration of the solutions were: $[(TPA)Fe(OTf)_2]$ (1 mmolar), ArIO (1.4 mmolar) and 1-methylcyclohexene (1 molar).

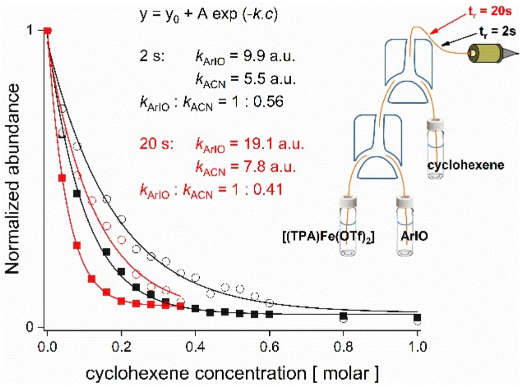

Figure S5. The relative abundance of $[(TPA)Fe^{IV}O(ArIO)]^{2+}$ (filled squares) and $[(TPA)Fe^{IV}O(MeCN)]^{2+}$ (empty cicles) in reaction with cyclohexene as a function of increasing concentration of cyclohexene. The reaction residence times (t_r) were set to 20 seconds (red) and 2 seconds (black) by changing the capillary length. The data are fitted with the exponential functions for a qualitative comparison. The derived rate constants do not reflect the rate constants of the C-H activation reactions initiated by either $[(TPA)Fe^{IV}O(ArIO)]^{2+}$ or $[(TPA)Fe^{IV}O(MeCN)]^{2+}$, because the data reflect not only the HAT reactions, but also the equilibration between both complexes.

Gas-phase reactivity

Gas phase reactivity of mass selected ions were measured at zero collision energy. The manifold temperature of the TSQ machine was kept constant at 70ºC. The reaction absorbance A_i for i-th reaction channel corresponding to the reaction P → F1 + F2 +…+ F_n, where P is reactant (parent ion) and F1…F_n are fragment ions with corresponding intensities I_P and I1…I_n is determined as: $A_i = -\ln(1-\Sigma I_n / (\Sigma I_n + I_P))\, I_i / \Sigma I_n$ .The dependence of A_i with the pressure of the neutral reactants 1,4-cyclohexadiene is plotted in Figure S5.

For figure 1E, gas phase reactivities were plotted as a function of the $[(TPA)FeO(X)]^{+/2+}$ decay. The y axis corresponds to the sum of the normalized intensities of parent and fragment ions that do not correspond to hydrogen atom transfer reactivity: $y = (\Sigma I_n + I_p – \Sigma I_{HAT})/(\Sigma I_n + I_p)$.

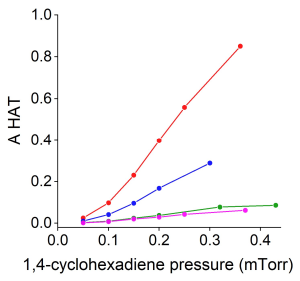

Figure S6. Dependence of reaction absorbance (A_{HAT}) for the reaction of $[(TPA)FeO(X)]^{+/2+}$ (X = ArI, ArIO, MeCN and TfO^-) uppon gas-phase collisions with 1,4-cyclohexadiene at mild zero-collision energy.

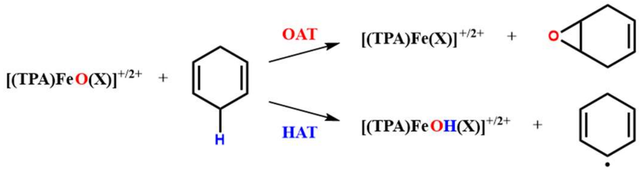

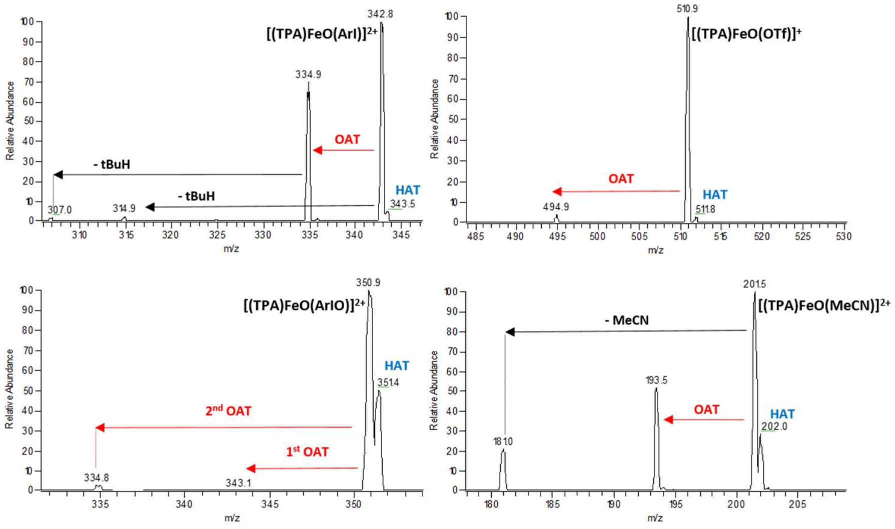

Figure S7. Gas phase reactivity of $[(TPA)FeO(X)]^{+/2+}$ (X = ArI, ArIO, MeCN and TfO^-) upon collisions with 1,4-cyclohexadiene at 0.2 mTorr and mild zero-collision energy. Blue stands for hydrogen atom transfer and red for oxygen atom transfer. Black arrows represent endothermic reactions as observed via CID.

Collision induced dissociation (CID) experiments with mass selected ions

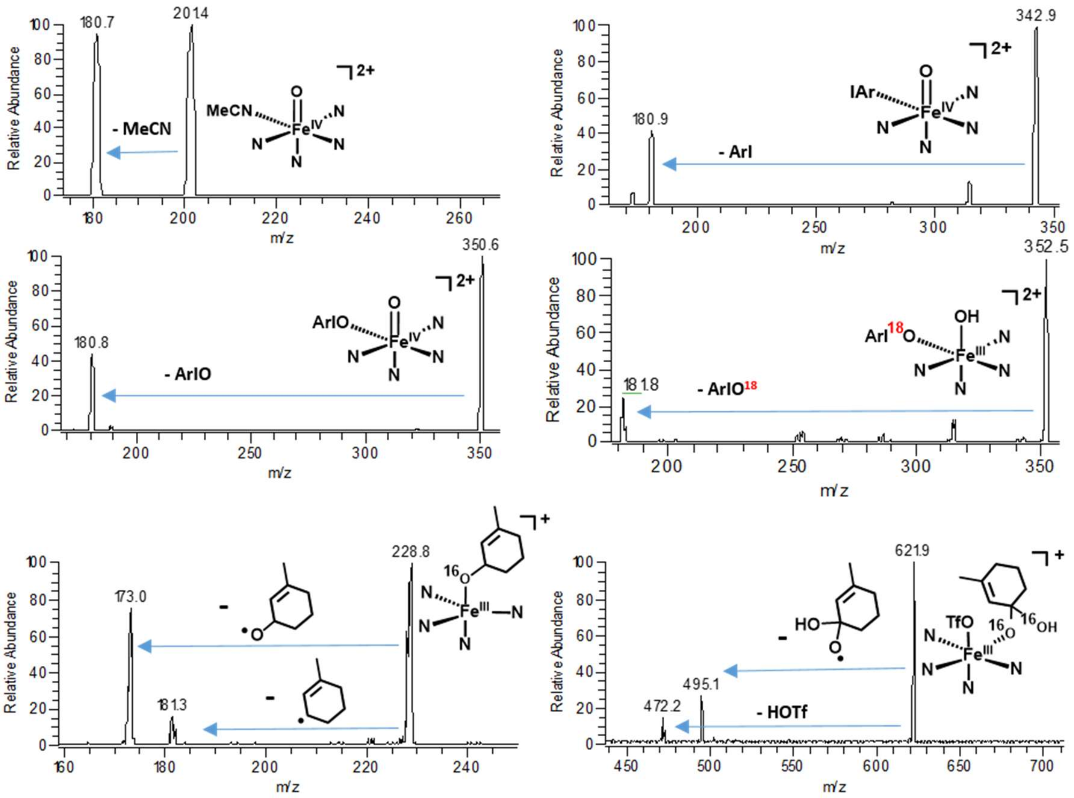

Figure S8. Collision induced dissociation (CID) of mass selected iron complexes containing the products from the reaction of [(TPA)FeO$^{16/18}$(X)]$^{n+}$ with Me-cyclohexene upon collisions with xenon gas.

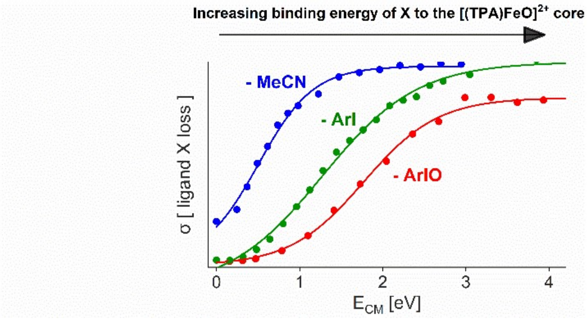

Figure S9. Energy dependence of the fragmentation yield (σ) for the loss of X in collision induced dissociation of [(TPA)FeO(X)]$^{+/2+}$ (X = CH_3CN, ArI, ArIO). The energy dependence for the loss of triflate anion could not be obtained because the elimination of an anion fragment from a cation does not happen in the gas phase.

Infrared photodissociation spectra

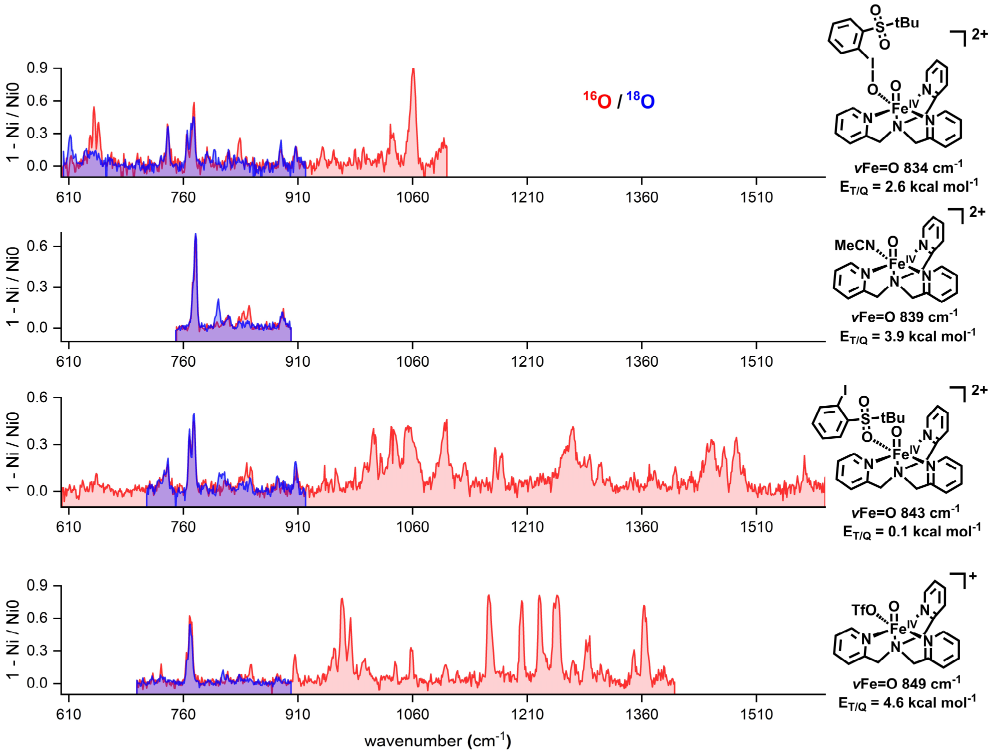

Figure S10. IRPD spectra of mass selected ions $[(TPA)Fe^{16}O(X)]^{n+}$ and $[(TPA)Fe^{18}O(X)]^{n+}$ generated via mixing of $[(TPA)Fe(OTf)_2]$ with $ArI^{16}O$ and $ArI^{18}O$, respectively. $E_{T/Q}$ is the DFT energy gap between the triplet and quintet spin states.

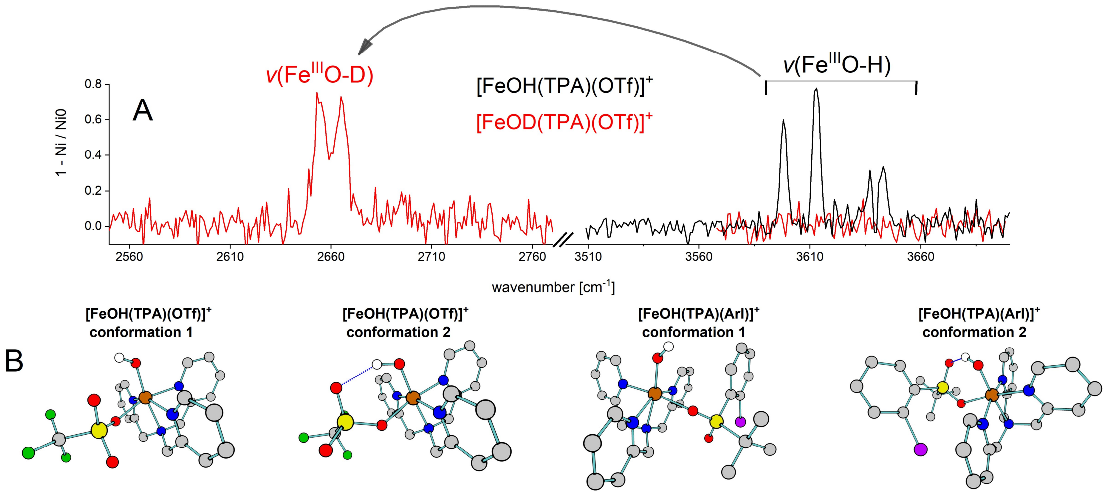

Figure S11. A) IRPD spectra of $[(TPA)Fe(OH)(OTf)]^{+}$ and $[(TPA)Fe(OD)(OTf)]^{+}$. B) DFT optimized structure for different conformers of the complexes $[(TPA)Fe(OH)(OTf)]^{+}$ and $[(TPA)Fe(OH)(ArI)]^{2+}$.

DFT calculations

Table S1. Selected parameters of complexes $[(TPA)FeO(X)]^{+/2+}$ obtained from DFT calculations at the b3lyp-d3/def2tzvp level of theory. Reported values are in the gas phase (without solvation model).

$[(TPA)Fe^{IV}O(X)]^{+/2+}$	Fe=O distance [Å]	Unscaled *v*(Fe=O) [cm^{-1}]	Charge transfer from X[a]	Triplet-quintet energy gap [$kcal.mol^{-1}$][b]
X = ArIO	1.624	934.5	+0.37	2.6
X = MeCN	1.626	913.7	+0.50	3.9
X = ArI	1.623	932.0	+0.28	0.1
X = TfO^-	1.621	932.5	+0.30	4.6

[a] The charge transfer was calculated as the difference between the charge of the free ligand X (-1 for TfO^- and 0 for ArIO, ArI and MeCN) and the sum of the charges at the atoms of ligand X when bounded to the core $[(TPA)Fe^{IV}O]$. [b] The calculations predict that all complexes have the triplet ground state.

Table S2. Selected parameters of complexes $[(TPA)FeOH(X)]^{+/2+}$ obtained from DFT calculations at the b3lyp-d3/def2tzvp level of theory. Reported values are in the gas phase (without solvation model).

$[(TPA)Fe^{III}OH(X)]^{+/2+}$	FeO-H distance [Å]	Unscaled *v*(FeO-H) [cm^{-1}]	Charge transfer from X[a]	Sextet-doublet energy gap [$kcal.mol^{-1}$][b]	Fe-O-H bond angle
X = ArIO	0.961	3851	+0.36	6.5	135^0
X = MeCN	0.961	3858	+0.29	2.1	145^0
X = ArI	0.961	3858	+0.19	6.5	137^0
X = TfO^-	0.968	3764	+0.43	5.4	119^0

[a] The charge transfer was calculated as the difference between the charge of the free ligand X (-1 for TfO^- and 0 for ArIO, ArI and MeCN) and the sum of the charges at the atoms of ligand X when bounded to the core $[(TPA)Fe^{III}OH]$. [b] The calculations predict that all complexes have the sextet ground state and the quartet spin state to be higher in energy.

Table S3. Relative free energies associated to the equilibrium reactions between $[(TPA)FeO(X)]^{n+}$ (X = ArIO, ArI, MeCN and TfO^-) calculated at the B3LYP-d3/def2tzvp level using SMD to account for solvation by acetonitrile.

**$[(TPA)Fe^{IV}O(X)]^{+/2+}$**	**Relative free energies $\Delta\Delta G^{298K}$ [$kJ.mol^{-1}$]**	**Equilibrium constants[a] for the equilibrium reaction with $[(TPA)Fe^{IV}O(MeCN)]^{2+}$ at 233 K[a]**	**Concentration of the ligands in solution[b] [mM]**	**k'_{rel} Relative rates to form for the given iron(IV)oxo complex from $[(TPA)Fe^{IV}O(MeCN)]^{2+}$ including the effect of the concentration of X**
**X = MeCN**	0	1	19147	1
**X = TfO^-**	-8.45	79	2	0.008
**X = ArI**	9.55	0.007	1	4.10^{-7}
**X = ArIO**	-52.99	7.7_x10^{11}	0.4	2.10^7

[a] Equilibrium constants were obtained by the formula K=exp(-ΔG/RT), where ΔG is the difference in free energy between two complexes, R is the universal gas constant and T is the temperature (233 K).

[b] Concentrations of the ligands were assumed for a reaction of $[(TPA)Fe(TfO)_2]$ (1 mM) in acetonitrile with 1.4 equivalents of ArIO. We assumed complete reaction leading to 1 mM solution of $[(TPA)FeO(X)]^{n+}$ with 2 equiv. of free TfO, 1 equiv. of ArI and 0.4 equiv. of ArIO available in solution.

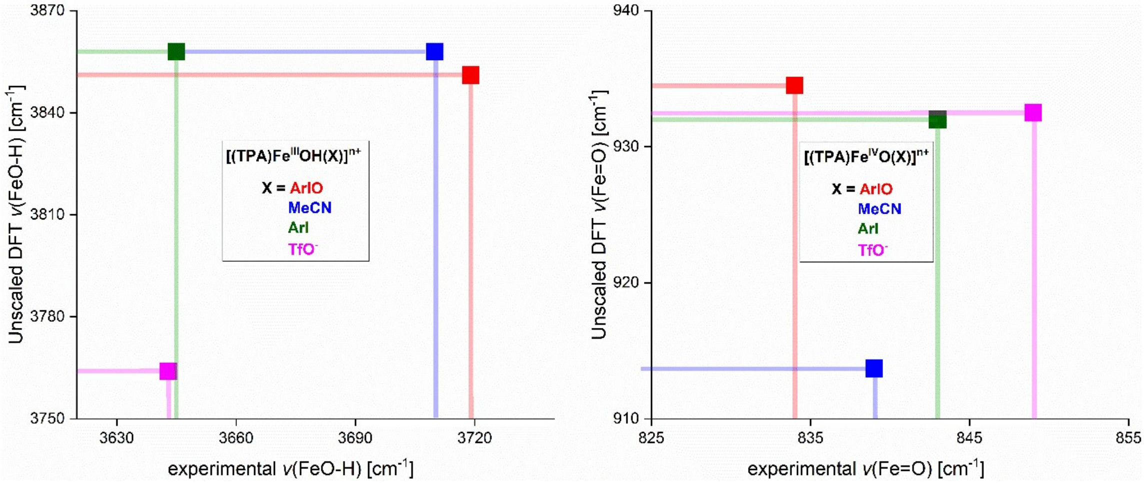

Figure S12. Experimental and theoretical Fe^{III}O-H (left) and Fe^{IV}=O (right) vibration frequencies for $[(TPA)FeO(X)]^{n+}$ and $[(TPA)FeOH(X)]^{n+}$, respectively.

XYZ coordinates from DFT calculations (gas phase).

$[(TPA)FeO(MeCN)]^{2+}$ S = 2

Fe	0.206480000	0.259278000	-0.594044000
N	2.121330000	-0.403098000	-0.291596000
N	-0.763455000	-1.630246000	-0.597002000
N	-1.627278000	1.111218000	-0.194978000
N	0.146756000	-0.091176000	1.514245000
O	0.230915000	0.416984000	-2.201153000
C	1.542948000	0.130555000	1.993200000
C	2.517876000	-0.446529000	0.998745000
C	3.764368000	-0.936747000	1.343495000
C	4.619283000	-1.373765000	0.335106000
C	4.202564000	-1.313782000	-0.989024000
C	2.938564000	-0.823002000	-1.267277000
C	-0.264945000	-1.512255000	1.763819000
C	-1.019173000	-2.135036000	0.619304000
C	-1.851379000	-3.230487000	0.784260000
C	-2.409960000	-3.822664000	-0.343310000
C	-2.125261000	-3.301562000	-1.600568000
C	-1.302137000	-2.192671000	-1.687823000
C	-0.822053000	0.895719000	2.074991000
C	-1.947371000	1.176748000	1.112524000
C	-3.211123000	1.569595000	1.517443000
C	-4.150510000	1.916754000	0.550905000
C	-3.802498000	1.857751000	-0.793146000
C	-2.526733000	1.440474000	-1.131232000
H	1.692167000	-0.284050000	2.992258000
H	1.707665000	1.206146000	2.048997000
H	4.064973000	-0.974909000	2.381945000
H	5.599200000	-1.759585000	0.584111000
H	-0.831300000	-1.579113000	2.693852000
H	0.645951000	-2.096908000	1.905437000

H	-2.052352000	-3.620308000	1.773303000
H	-3.060318000	-4.681379000	-0.240084000
H	-0.281236000	1.830187000	2.229060000
H	-1.198715000	0.570161000	3.046114000
H	-3.456796000	1.610742000	2.570132000
H	-5.143628000	2.228616000	0.846729000
H	2.547248000	-0.749907000	-2.272673000
H	4.841068000	-1.644038000	-1.796170000
H	-1.056685000	-1.720762000	-2.629539000
H	-2.538670000	-3.739271000	-2.498230000
H	-4.506035000	2.122994000	-1.569637000
H	-2.191107000	1.356106000	-2.155849000
C	1.448887000	3.454390000	-0.458210000
N	1.058903000	2.407510000	-0.191174000
C	1.940604000	4.773490000	-0.800306000
H	2.934628000	4.921450000	-0.375721000
H	1.997112000	4.870173000	-1.885930000
H	1.265547000	5.535247000	-0.407583000

[(TPA)FeO(MeCN)]2+ S = 1

Fe	0.002623000	-0.358301000	-0.539632000
N	-1.965445000	-0.358358000	-0.244796000
N	-0.008328000	1.613986000	-0.487382000
N	1.970784000	-0.339865000	-0.245637000
N	0.002704000	-0.163796000	1.563985000
O	0.002567000	-0.402351000	-2.164970000
C	-1.244143000	-0.843082000	2.006297000
C	-2.354333000	-0.531950000	1.034733000
C	-3.691795000	-0.482621000	1.386896000
C	-4.643500000	-0.267334000	0.394939000
C	-4.228614000	-0.103522000	-0.920136000
C	-2.873826000	-0.150067000	-1.205205000
C	-0.001858000	1.288757000	1.922324000
C	-0.012131000	2.193904000	0.721209000
C	-0.023179000	3.574802000	0.856849000
C	-0.029920000	4.367258000	-0.281053000
C	-0.025458000	3.754217000	-1.529201000
C	-0.014844000	2.374716000	-1.596316000
C	1.254940000	-0.833936000	2.005134000
C	2.361972000	-0.512213000	1.033394000
C	3.699157000	-0.451855000	1.384835000
C	4.648353000	-0.226647000	0.392664000
C	4.231254000	-0.064205000	-0.921870000
C	2.876742000	-0.122098000	-1.206229000
H	-1.514088000	-0.570548000	3.028447000
H	-1.061938000	-1.918051000	1.991061000
H	-3.984740000	-0.614112000	2.419921000
H	-5.694537000	-0.226651000	0.648768000
H	0.874528000	1.513015000	2.532206000
H	-0.872364000	1.505633000	2.543178000
H	-0.026214000	4.018055000	1.844058000
H	-0.038504000	5.445857000	-0.196220000
H	1.081285000	-1.910276000	1.988498000
H	1.523107000	-0.560579000	3.027541000
H	3.993860000	-0.582464000	2.417472000

H	5.699153000	-0.177265000	0.645933000
H	-2.482795000	-0.027572000	-2.205334000
H	-4.938191000	0.062756000	-1.718364000
H	-0.011386000	1.839349000	-2.534226000
H	-0.030312000	4.332613000	-2.442241000
H	4.938870000	0.109593000	-1.720237000
H	2.484094000	-0.001526000	-2.205958000
C	0.017006000	-3.489894000	-0.654591000
N	0.012029000	-2.363211000	-0.446161000
C	0.023279000	-4.909921000	-0.934914000
H	-0.865009000	-5.378397000	-0.508344000
H	0.023933000	-5.061424000	-2.016211000
H	0.915687000	-5.370530000	-0.508374000

[(TPA)FeO(OTf)]2+ S = 1

O	1.435600000	0.219332000	-0.052242000
S	2.483420000	-0.876991000	-0.113552000
O	2.387621000	-1.691663000	-1.289304000
C	3.993583000	0.200110000	-0.310779000
F	4.081491000	1.051160000	0.719820000
F	5.079611000	-0.563877000	-0.338209000
F	3.916063000	0.901776000	-1.440667000
O	2.618988000	-1.519874000	1.170928000
Fe	-0.460515000	0.146847000	-0.488306000
N	-0.514195000	-1.801450000	-0.149222000
N	-2.454607000	0.111326000	-0.572974000
N	-0.387357000	2.120409000	-0.231219000
N	-0.851345000	0.241782000	1.608441000
O	-0.321808000	0.119304000	-2.102733000
C	-0.365923000	-1.053327000	2.149130000
C	-0.544951000	-2.163096000	1.146401000
C	-0.608456000	-3.497897000	1.508806000
C	-0.612708000	-4.467293000	0.514509000
C	-0.553110000	-4.077133000	-0.816707000
C	-0.505621000	-2.727632000	-1.114501000
C	-2.306898000	0.450546000	1.816995000
C	-3.119460000	0.145077000	0.589917000
C	-4.495954000	-0.022434000	0.639783000
C	-5.196528000	-0.212183000	-0.542113000
C	-4.498220000	-0.230551000	-1.743313000
C	-3.124763000	-0.072649000	-1.720222000
C	-0.069276000	1.419680000	2.062121000
C	-0.194850000	2.521678000	1.039442000
C	-0.060731000	3.865147000	1.348004000
C	-0.103218000	4.801354000	0.321675000
C	-0.285746000	4.369102000	-0.985000000
C	-0.426207000	3.012442000	-1.225002000
H	-0.845699000	-1.294685000	3.100148000
H	0.707762000	-0.968233000	2.320995000
H	-0.628480000	-3.770868000	2.554983000
H	-0.648820000	-5.515968000	0.778037000
H	-2.473805000	1.500161000	2.067471000
H	-2.661485000	-0.133202000	2.668736000
H	-5.006006000	0.000217000	1.593611000
H	-6.270139000	-0.345099000	-0.526011000

H	0.977550000	1.124952000	2.110985000
H	-0.381644000	1.756344000	3.053831000
H	0.085799000	4.169907000	2.375407000
H	0.008136000	5.854895000	0.541278000
H	-0.433523000	-2.349830000	-2.123414000
H	-0.531074000	-4.802256000	-1.617308000
H	-2.516661000	-0.094447000	-2.612963000
H	-5.003483000	-0.373667000	-2.687818000
H	-0.315034000	5.065613000	-1.810680000
H	-0.555658000	2.601575000	-2.216245000

[(TPA)FeO(OTf)]2+ S = 2

O	1.372512000	0.590665000	0.029252000
S	2.764712000	0.003203000	-0.096137000
O	2.933216000	-0.781933000	-1.284871000
C	3.744816000	1.571128000	-0.336366000
F	3.558767000	2.387566000	0.707787000
F	5.036627000	1.279047000	-0.437078000
F	3.344412000	2.196147000	-1.444796000
O	3.187088000	-0.545107000	1.168703000
Fe	-0.408148000	-0.118645000	-0.565577000
N	0.233516000	-2.073923000	-0.210076000
N	-2.476216000	-0.635683000	-0.669000000
N	-1.117552000	1.826281000	-0.126168000
N	-0.856818000	-0.339146000	1.542857000
O	-0.270852000	-0.051950000	-2.170258000
C	0.166650000	-1.281537000	2.074261000
C	0.429408000	-2.386043000	1.083513000
C	0.951287000	-3.614891000	1.449590000
C	1.298555000	-4.519516000	0.453598000
C	1.112951000	-4.174808000	-0.879134000
C	0.575542000	-2.935182000	-1.174625000
C	-2.226154000	-0.902749000	1.720040000
C	-3.104012000	-0.772190000	0.504393000
C	-4.485955000	-0.877014000	0.575461000
C	-5.220558000	-0.859295000	-0.602936000
C	-4.556014000	-0.734883000	-1.817635000
C	-3.177232000	-0.615828000	-1.808793000
C	-0.716327000	1.025742000	2.116035000
C	-1.253703000	2.082024000	1.184225000
C	-1.764546000	3.292012000	1.627035000
C	-2.115422000	4.255855000	0.688400000
C	-1.950733000	3.984087000	-0.663860000
C	-1.450671000	2.746371000	-1.034234000
H	-0.130135000	-1.678038000	3.048409000
H	1.102966000	-0.738556000	2.200123000
H	1.106059000	-3.848300000	2.494136000
H	1.721760000	-5.479849000	0.716939000
H	-2.707896000	-0.461204000	2.594989000
H	-2.123142000	-1.970239000	1.924767000
H	-4.975429000	-0.977036000	1.535126000
H	-6.299141000	-0.941055000	-0.573027000
H	0.351372000	1.214993000	2.228230000
H	-1.180674000	1.090286000	3.103017000
H	-1.873516000	3.481226000	2.686450000

H	-2.509761000	5.210291000	1.011494000
H	0.428335000	-2.587854000	-2.187401000
H	1.391334000	-4.845614000	-1.679067000
H	-2.593652000	-0.492760000	-2.711251000
H	-5.093457000	-0.721078000	-2.755177000
H	-2.203022000	4.713630000	-1.420180000
H	-1.297332000	2.464016000	-2.067327000

[(TPA)FeO(ArI)]2+ S = 2

Fe	-1.798025000	-0.056492000	-0.541259000
C	1.019044000	-3.311269000	-0.472262000
C	1.166654000	-3.538162000	0.892034000
C	0.465505000	-2.750572000	1.795057000
C	-0.353970000	-1.742343000	1.315002000
N	-0.483021000	-1.529598000	-0.007751000
C	0.178329000	-2.298226000	-0.887202000
C	-4.181831000	3.471573000	-1.217797000
C	-4.291084000	3.990085000	0.066821000
C	-3.689161000	3.322142000	1.129097000
C	-2.988101000	2.156255000	0.876873000
N	-2.897239000	1.660228000	-0.374565000
C	-3.474015000	2.296675000	-1.402033000
C	-4.556996000	-3.358006000	-1.322443000
C	-5.049557000	-3.625987000	-0.050502000
C	-4.688685000	-2.801163000	1.009992000
C	-3.847058000	-1.729394000	0.762672000
N	-3.364152000	-1.488733000	-0.465973000
C	-3.704236000	-2.280467000	-1.490737000
C	-3.499354000	-0.698841000	1.800249000
C	-2.235007000	1.391910000	1.934814000
C	-1.049909000	-0.773305000	2.233312000
N	-2.170881000	-0.050462000	1.558885000
H	1.821728000	-4.321423000	1.249494000
H	0.565986000	-2.904045000	2.860895000
H	-4.843132000	4.903929000	0.243396000
H	-3.763964000	3.702462000	2.138991000
H	-5.708221000	-4.468217000	0.116126000
H	-5.062469000	-2.983573000	2.008618000
H	-4.259748000	0.082648000	1.746247000
H	-3.538937000	-1.113258000	2.808576000
H	-2.682142000	1.524633000	2.922491000
H	-1.214360000	1.768814000	1.967327000
H	-0.304150000	-0.038939000	2.534976000
H	-1.404247000	-1.277216000	3.133583000
I	4.288609000	-0.787832000	0.888258000
C	3.248590000	-0.434654000	-0.919600000
C	2.118855000	0.379840000	-1.102478000
C	1.526448000	0.490220000	-2.367187000
H	0.639818000	1.093539000	-2.487372000
C	2.046445000	-0.191125000	-3.453291000
H	1.579414000	-0.094414000	-4.423848000
C	3.169112000	-0.991576000	-3.280632000
H	3.595297000	-1.525505000	-4.120130000
C	3.757273000	-1.113808000	-2.028993000
H	4.628461000	-1.742851000	-1.911177000

S	1.315438000	1.337754000	0.177854000
O	1.537913000	0.762288000	1.485370000
O	-0.109273000	1.444539000	-0.220518000
C	1.991698000	3.054300000	0.132774000
C	1.224462000	3.803441000	1.229615000
H	1.612880000	4.821418000	1.274978000
H	1.369119000	3.345118000	2.207297000
H	0.159228000	3.861570000	1.003942000
C	1.735033000	3.661862000	-1.245623000
H	0.674714000	3.662183000	-1.495810000
H	2.079936000	4.696486000	-1.226914000
H	2.288748000	3.145622000	-2.028535000
C	3.484914000	2.968014000	0.450666000
H	4.030948000	2.412192000	-0.311381000
H	3.885305000	3.982462000	0.469881000
H	3.667024000	2.509862000	1.421028000
H	-4.821057000	-3.975302000	-2.169458000
H	-3.266802000	-2.024814000	-2.446265000
H	0.020344000	-2.059129000	-1.928788000
H	1.547978000	-3.905084000	-1.203522000
H	-3.344340000	1.836982000	-2.372030000
H	-4.637502000	3.964383000	-2.064905000
O	-1.634123000	-0.117484000	-2.148814000

[(TPA)FeO(ArI)]2+ S = 1

Fe	1.669047000	-0.099351000	0.411182000
C	-1.055591000	-3.350202000	0.366290000
C	-1.229045000	-3.565345000	-0.995998000
C	-0.548199000	-2.763874000	-1.901471000
C	0.277730000	-1.757967000	-1.426525000
N	0.434905000	-1.560542000	-0.103769000
C	-0.208510000	-2.339768000	0.778068000
C	4.317899000	3.125677000	1.249930000
C	4.471783000	3.698466000	-0.005821000
C	3.828979000	3.125783000	-1.098442000
C	3.043948000	2.002367000	-0.904247000
N	2.908946000	1.454517000	0.320604000
C	3.525503000	1.996830000	1.376351000
C	4.379894000	-3.132510000	1.551607000
C	4.991172000	-3.460869000	0.347622000
C	4.654486000	-2.747014000	-0.793761000
C	3.720616000	-1.726571000	-0.701860000
N	3.124755000	-1.428828000	0.463523000
C	3.443951000	-2.115393000	1.572875000
C	3.384777000	-0.836980000	-1.861722000
C	2.261358000	1.330993000	-2.005538000
C	0.954743000	-0.771228000	-2.341040000
N	2.099660000	-0.106702000	-1.662762000
H	-1.887158000	-4.347891000	-1.349197000
H	-0.666523000	-2.903775000	-2.967353000
H	5.088980000	4.577488000	-0.136778000
H	3.936612000	3.544559000	-2.090053000
H	5.718812000	-4.260050000	0.298105000
H	5.113800000	-2.970664000	-1.747409000
H	4.186654000	-0.102219000	-1.955180000

H	3.365741000	-1.399706000	-2.796056000
H	2.742919000	1.463783000	-2.977142000
H	1.269736000	1.776560000	-2.056012000
H	0.216578000	-0.011424000	-2.592284000
H	1.269257000	-1.249392000	-3.270235000
I	-4.315853000	-0.659331000	-0.832587000
C	-3.229973000	-0.346742000	0.955037000
C	-2.058480000	0.413151000	1.120249000
C	-1.458337000	0.525644000	2.380974000
H	-0.546466000	1.091289000	2.488394000
C	-2.004339000	-0.110828000	3.480904000
H	-1.530264000	-0.016171000	4.448127000
C	-3.160990000	-0.864965000	3.325713000
H	-3.606256000	-1.365154000	4.176023000
C	-3.761657000	-0.981620000	2.079332000
H	-4.662858000	-1.569864000	1.978019000
S	-1.230349000	1.311514000	-0.179046000
O	-1.544712000	0.775164000	-1.481381000
O	0.226237000	1.322652000	0.155347000
C	-1.751371000	3.082444000	-0.098721000
C	-0.971702000	3.774425000	-1.223456000
H	-1.296453000	4.814678000	-1.263104000
H	-1.175282000	3.323571000	-2.194064000
H	0.101040000	3.767201000	-1.029099000
C	-1.398407000	3.665272000	1.268350000
H	-0.334819000	3.572889000	1.485384000
H	-1.650506000	4.726427000	1.254244000
H	-1.971838000	3.203158000	2.070164000
C	-3.258830000	3.105507000	-0.360450000
H	-3.814712000	2.584375000	0.418895000
H	-3.583902000	4.146644000	-0.358064000
H	-3.510515000	2.670760000	-1.326141000
H	4.615120000	-3.658874000	2.465785000
H	2.922960000	-1.822960000	2.472110000
H	-0.033806000	-2.111926000	1.819153000
H	-1.568256000	-3.952443000	1.102400000
H	3.355699000	1.504686000	2.323402000
H	4.803043000	3.540853000	2.121951000
O	1.470584000	-0.145337000	2.020843000

[(TPA)FeO(ArIO)]2+ S = 1

Fe	-1.806401000	0.052367000	0.153966000
N	-2.118817000	1.962186000	-0.315887000
N	-3.601188000	-0.021653000	1.042051000
N	-1.812710000	-1.935492000	0.129336000
N	-2.985242000	-0.220339000	-1.595204000
O	-0.996568000	0.279250000	1.542965000
C	-2.630411000	0.947954000	-2.442671000
C	-2.547944000	2.177283000	-1.574234000
C	-2.843775000	3.455747000	-2.017712000
C	-2.683099000	4.527080000	-1.146085000
C	-2.239588000	4.288516000	0.148836000
C	-1.970088000	2.985465000	0.532425000
C	-4.431771000	-0.225807000	-1.232809000
C	-4.668557000	-0.207497000	0.254806000

C	-5.943247000	-0.342150000	0.787679000
C	-6.111496000	-0.273865000	2.162566000
C	-4.996818000	-0.073662000	2.969524000
C	-3.755217000	0.044644000	2.374477000
C	-2.527143000	-1.521830000	-2.141446000
C	-2.257886000	-2.488860000	-1.016257000
C	-2.383371000	-3.862532000	-1.140588000
C	-2.028262000	-4.674170000	-0.068253000
C	-1.562494000	-4.088910000	1.102209000
C	-1.473227000	-2.708503000	1.167892000
H	-3.334716000	1.082775000	-3.266388000
H	-1.641111000	0.763150000	-2.859451000
H	-3.195413000	3.608904000	-3.029106000
H	-2.909508000	5.533402000	-1.472883000
H	-4.923697000	-1.093634000	-1.675316000
H	-4.909974000	0.653040000	-1.668943000
H	-6.789006000	-0.495660000	0.130303000
H	-7.096569000	-0.375740000	2.598303000
H	-1.586155000	-1.341656000	-2.659840000
H	-3.237911000	-1.933807000	-2.860846000
H	-2.750598000	-4.289990000	-2.063935000
H	-2.118075000	-5.749589000	-0.146674000
O	-0.261099000	0.072910000	-1.028973000
I	1.384459000	-0.761216000	-0.505379000
C	2.298191000	1.021610000	0.241717000
C	3.579906000	0.990147000	0.772666000
C	4.099124000	2.140284000	1.363842000
H	5.073384000	2.106998000	1.831373000
C	3.350928000	3.309939000	1.365437000
H	3.755592000	4.203388000	1.820952000
C	2.086373000	3.329424000	0.787675000
H	1.508914000	4.244634000	0.780839000
C	1.540679000	2.175588000	0.231293000
H	0.549121000	2.167502000	-0.193813000
S	4.542249000	-0.516057000	0.759838000
O	3.571787000	-1.517998000	0.252239000
O	5.126664000	-0.719268000	2.054160000
C	5.861538000	-0.370058000	-0.520579000
C	6.536450000	-1.749857000	-0.522848000
H	7.329559000	-1.729619000	-1.271492000
H	5.837848000	-2.542410000	-0.787885000
H	6.985473000	-1.977590000	0.442915000
C	6.842247000	0.723898000	-0.095859000
H	7.225960000	0.552165000	0.908954000
H	7.686819000	0.705781000	-0.785939000
H	6.397552000	1.717111000	-0.152118000
C	5.200736000	-0.058456000	-1.862925000
H	4.673573000	0.896811000	-1.847330000
H	5.982968000	0.011708000	-2.619791000
H	4.512332000	-0.846746000	-2.164906000
H	-1.621188000	2.724585000	1.521893000
H	-2.111578000	5.094621000	0.857462000
H	-2.847169000	0.192820000	2.940729000
H	-5.083201000	-0.013249000	4.045172000
H	-1.276371000	-4.686429000	1.956173000
H	-1.123964000	-2.184088000	2.046309000

[(TPA)FeO(ArIO)]2+ S = 2

Fe	-1.703472000	0.063883000	0.265207000
N	-1.993859000	2.198152000	-0.315845000
N	-3.612059000	0.122021000	1.131095000
N	-2.143145000	-2.127123000	0.035100000
N	-2.964650000	-0.025779000	-1.554507000
O	-0.933904000	0.128451000	1.687807000
C	-2.499568000	1.102352000	-2.401712000
C	-2.408541000	2.365819000	-1.581656000
C	-2.690325000	3.625674000	-2.087442000
C	-2.529835000	4.732121000	-1.259999000
C	-2.102758000	4.547095000	0.049603000
C	-1.847877000	3.256857000	0.486806000
C	-4.401790000	0.125943000	-1.178970000
C	-4.664715000	0.049149000	0.304190000
C	-5.958285000	-0.042424000	0.799095000
C	-6.158328000	-0.045613000	2.172483000
C	-5.058527000	0.039568000	3.019469000
C	-3.797129000	0.116040000	2.460095000
C	-2.681259000	-1.342477000	-2.179765000
C	-2.680308000	-2.447291000	-1.152329000
C	-3.135425000	-3.729847000	-1.415981000
C	-3.013824000	-4.701216000	-0.426972000
C	-2.449404000	-4.360898000	0.795712000
C	-2.031043000	-3.053119000	0.990872000
H	-3.143047000	1.239187000	-3.274598000
H	-1.497672000	0.848455000	-2.750153000
H	-3.030947000	3.739818000	-3.107888000
H	-2.744932000	5.725295000	-1.631908000
H	-5.002113000	-0.623017000	-1.698684000
H	-4.752926000	1.098072000	-1.530139000
H	-6.795500000	-0.106753000	0.116518000
H	-7.159600000	-0.115477000	2.576797000
H	-1.679428000	-1.279919000	-2.605638000
H	-3.382562000	-1.553813000	-2.990779000
H	-3.573317000	-3.967239000	-2.376368000
H	-3.360228000	-5.709937000	-0.609696000
O	-0.174622000	-0.115733000	-0.878801000
I	1.505314000	-0.912392000	-0.353247000
C	2.411106000	0.925840000	0.247950000
C	3.714240000	0.934764000	0.724800000
C	4.246194000	2.119229000	1.230192000
H	5.239106000	2.119914000	1.657869000
C	3.485363000	3.280390000	1.201137000
H	3.898916000	4.200806000	1.590070000
C	2.196047000	3.256496000	0.681335000
H	1.607076000	4.163885000	0.653299000
C	1.638603000	2.069728000	0.211997000
H	0.627499000	2.033958000	-0.162819000
S	4.681730000	-0.566649000	0.764101000
O	3.692224000	-1.594202000	0.338601000
O	5.300940000	-0.705352000	2.049705000
C	5.961553000	-0.498157000	-0.562473000
C	6.633527000	-1.878257000	-0.506688000
H	7.404112000	-1.901854000	-1.278390000

H	5.926592000	-2.682395000	-0.706701000
H	7.110777000	-2.053046000	0.456527000
C	6.954852000	0.615390000	-0.227109000
H	7.368066000	0.498605000	0.773885000
H	7.778764000	0.556397000	-0.939495000
H	6.509883000	1.605171000	-0.325982000
C	5.259908000	-0.259468000	-1.898836000
H	4.734494000	0.696666000	-1.920266000
H	6.019213000	-0.232998000	-2.681384000
H	4.562667000	-1.061847000	-2.136557000
H	-1.517642000	3.043435000	1.495364000
H	-1.978085000	5.382958000	0.723713000
H	-2.895341000	0.168122000	3.054495000
H	-5.172917000	0.040231000	4.094160000
H	-2.339562000	-5.088924000	1.587150000
H	-1.596200000	-2.719903000	1.924486000

References

[S1] D. Macikenas, E. Skrzypczak-Jankun, J. D. Protasiewicz, *J. Am. Chem. Soc.* **1999**, *121*, 7164–7165.
[S2] M. H. Lim, J.-U. Rohde, A. Stubna, M. R. Bukowski, M. Costas, R. Y. N. Ho, E. Munck, W. Nam, L. Que, Jr., *Proc. Natl. Acad. Sci.* **2003**, *100*, 3665–3670.
[S3] L. Ducháčková, J. Roithová, *Chem. - A Eur. J.* **2009**, *15*, 13399–13405.
[S4] J. Jašík, J. Žabka, J. Roithová, D. Gerlich, *Int. J. Mass Spectrom.* **2013**, *354–355*, 204–210.
[S5] Gaussian 16, Revision C.01, Frisch, M. J.; Trucks, G. W.; Schlegel, H. B.; Scuseria, G. E.; Robb, M. A.; Cheeseman, J. R.; Scalmani, G.; Barone, V.; Petersson, G. A.; Nakatsuji, H.; Li, X.; Caricato, M.; Marenich, A. V.; Bloino, J.; Janesko, B. G.; Gomperts, R.; Mennucci, B.; Hratchian, H. P.; Ortiz, J. V.; Izmaylov, A. F.; Sonnenberg, J. L.; Williams-Young, D.; Ding, F.; Lipparini, F.; Egidi, F.; Goings, J.; Peng, B.; Petrone, A.; Henderson, T.; Ranasinghe, D.; Zakrzewski, V. G.; Gao, J.; Rega, N.; Zheng, G.; Liang, W.; Hada, M.; Ehara, M.; Toyota, K.; Fukuda, R.; Hasegawa, J.; Ishida, M.; Nakajima, T.; Honda, Y.; Kitao, O.; Nakai, H.; Vreven, T.; Throssell, K.; Montgomery, J. A., Jr.; Peralta, J. E.; Ogliaro, F.; Bearpark, M. J.; Heyd, J. J.; Brothers, E. N.; Kudin, K. N.; Staroverov, V. N.; Keith, T. A.; Kobayashi, R.; Normand, J.; Raghavachari, K.; Rendell, A. P.; Burant, J. C.; Iyengar, S. S.; Tomasi, J.; Cossi, M.; Millam, J. M.; Klene, M.; Adamo, C.; Cammi, R.; Ochterski, J. W.; Martin, R. L.; Morokuma, K.; Farkas, O.; Foresman, J. B.; Fox, D. J. Gaussian, Inc., Wallingford CT, 2016.
